# THE ASSOCIATIONS BETWEEN LUNG DISEASE, CAREGIVING, AND PSYCHOSOCIAL WELL-BEING IN OLDER US ADULTS

**DOI:** 10.1093/geroni/igad104.3629

**Published:** 2023-12-21

**Authors:** Mariah Malak Bilalaga, Louay Almidani

**Affiliations:** Medstar Health, Baltimore, Maryland, United States; Wilmer Eye Institute, Baltimore, Maryland, United States

## Abstract

The aim of this study was to examine the association between lung disease, caregiving for adults with lung disease, and the psychological and social well-being of older adults using data from the National Health and Aging Trends Study 2021, a nationally representative sample of US adults aged ≥ 65 years. Lung disease was determined based on self-reported data regarding diagnoses of emphysema, asthma, or chronic bronchitis. The Patient Health Questionnaire for Depression and Anxiety (PHQ-4) questionnaire was utilized to assess depressive and anxiety symptoms. Social isolation was defined based on responses about living arrangements, frequency of communication with others, and participation in activities. Prevalence ratios adjusted for covariates were calculated using Poisson regression. Among adults with lung disease, the prevalence of depressive symptoms was notably higher (prevalence ratio [PR]: 1.43; 95% CI: 1.05, 1.96) compared to those without. However, the prevalence of anxiety symptoms and social isolation did not significantly differ when compared to their counterparts without lung disease (p< 0.05). Caregivers responsible for adults with lung disease had a greater prevalence of depressive symptoms (PR: 1.61; 95% CI: 1.16-2.25), but there was no notable difference in the prevalence of anxiety symptoms or social isolation (p< 0.05). We highlight that both lung disease and caregiving for adults with lung disease are significantly associated with increased prevalence of depressive symptoms, but not anxiety symptoms or social isolation, among older adults in the United States. Strategies are required to enhance the psychological well-being of individuals with lung disease as well as their caregivers.

